# Evaluation of the *Pichia pastoris *expression system for the production of GPCRs for structural analysis

**DOI:** 10.1186/1475-2859-10-24

**Published:** 2011-04-22

**Authors:** Hidetsugu Asada, Tomoko Uemura, Takami Yurugi-Kobayashi, Mitsunori Shiroishi, Tatsuro Shimamura, Hirokazu Tsujimoto, Keisuke Ito, Taishi Sugawara, Takanori Nakane, Norimichi Nomura, Takeshi Murata, Tatsuya Haga, So Iwata, Takuya Kobayashi

**Affiliations:** 1 Iwata Human Receptor Crystallography project, ERATO, JST, Konoe-cho, Yoshida, Sakyo-ku, Kyoto 606-8501, Japan; 2 Department of Medical Chemistry, Kyoto University Faculty of Medicine, Konoe-cho, Yoshida, Sakyo-ku, Kyoto 606-8501, Japan; 3 Department of Applied Biological Chemistry, Graduate School of Agricultural and Life Sciences, The University of Tokyo, Bunkyo-ku, Tokyo 113-8657, Japan; 4 Institute for Biomolecular Science, Faculty of Science, Gakushuin University, 1-5-1 Mejiro, Toshima-ku, Tokyo 171-8588, Japan; 5 RIKEN Genomic Science Center, 1-7-22 Suehiro-cho, Tsurumi, Yokohama 230-0045, Japan; 6 Membrane Protein Crystallography Group, Division of Molecular Biosciences, Imperial College London, SW7 2AZ, UK

## Abstract

**Background:**

Various protein expression systems, such as *Escherichia coli *(*E. coli*), *Saccharomyces cerevisiae *(*S. cerevisiae*), *Pichia pastoris *(*P. pastoris*), insect cells and mammalian cell lines, have been developed for the synthesis of G protein-coupled receptors (GPCRs) for structural studies. Recently, the crystal structures of four recombinant human GPCRs, namely β_2 _adrenergic receptor, adenosine A_2a _receptor, CXCR4 and dopamine D3 receptor, were successfully determined using an insect cell expression system. GPCRs expressed in insect cells are believed to undergo mammalian-like posttranscriptional modifications and have similar functional properties than in mammals. Crystal structures of GPCRs have not yet been solved using yeast expression systems. In the present study, *P. pastoris *and insect cell expression systems for the human muscarinic acetylcholine receptor M2 subtype (CHRM2) were developed and the quantity and quality of CHRM2 synthesized by both expression systems were compared for the application in structural studies.

**Results:**

The ideal conditions for the expression of CHRM2 in *P. pastoris *were 60 hr at 20°C in a buffer of pH 7.0. The specific activity of the expressed CHRM2 was 28.9 pmol/mg of membrane protein as determined by binding assays using [^3^H]-quinuclidinyl benzilate (QNB). Although the specific activity of the protein produced by *P. pastoris *was lower than that of Sf9 insect cells, CHRM2 yield in *P. pastoris *was 2-fold higher than in Sf9 insect cells because *P. pastoris *was cultured at high cell density. The dissociation constant (Kd) for QNB in *P. pastoris *was 101.14 ± 15.07 pM, which was similar to that in Sf9 insect cells (86.23 ± 8.57 pM). There were no differences in the binding affinity of CHRM2 for QNB between *P. pastoris *and Sf9 insect cells.

**Conclusion:**

Compared to insect cells, *P. pastoris *is easier to handle, can be grown at lower cost, and can be expressed quicker at a large scale. Yeast, *P. pastoris*, and insect cells are all effective expression systems for GPCRs. The results of the present study strongly suggested that protein expression in *P. pastoris *can be applied to the structural and biochemical studies of GPCRs.

## Background

G protein-coupled receptors (GPCRs) belong to the largest superfamily of cell surface receptors. The GPCRs are integral transmembrane proteins and mediate various cellular responses to specific functional ligands including amine, eicosanoid, hormone and peptide, as well as taste and light stimuli. Approximately 50% of all currently available drugs act through GPCRs [1,2]. GPCRs are among the most important therapeutic targets for various disorders. Structure guided drug development is therefore important for the design of novel drugs devoid of side effects.

The crystal structure of membrane proteins such as GPCRs is difficult to solve due to several technical bottlenecks. One of the main obstacles for the resolution of crystal structures is the preparation of sufficiently large amounts of functional GPCR protein [3]. Milligram quantities of purified protein are required for crystallization and structural determination. Bovine rhodopsin [4], bovine opsin [5] and squid rhodopsin [6,7], whose crystal structures have been solved, are expressed in large amounts endogenously, and therefore can be obtained from natural sources, whereas other GPCRs cannot be purified in large amounts from natural tissues because of their low endogenous expression levels. In addition to insect cells [8-16], various expression systems can be applied to obtain a high yield of GPCRs, such as *Escherichia coli *[17-21], *Saccharomyces cerevisiae *[22,23], *Pichia pastoris *[24-30], mammalian cell lines [31-33], and a cell-free translation system [34-39]. We previously identified 25 GPCRs expressed by *P. pastoris *[29], which led us to suggest that *P. pastoris *is a suitable host for GPCR crystal structural studies. However, the crystal structures of recombinant human β_2_-adrenergic receptor (ADRB2) [40,41], human adenosine A_2A _receptor (ADORA2A) [42], human CXCR4 chemokine receptor [43], human dopamine D3 receptor [44] and turkey β_1_-adrenergic receptor (ADRB1) [13] have been determined successfully using only insect cells, in the present study, the quality and quantity of GPCRs expressed in *P. pastoris *were compared directly with those from Sf9 insect cells. Together with our previous study, the results show clearly that the *P. pastoris *expression system is an efficient system for GPCR production.

The muscarinic acetylcholine receptor (CHRM) belongs to the GPCR superfamily and plays important roles in signal transduction in the central and peripheral nervous systems [45,46]. The muscarinic actions of acetylcholine in central and peripheral physiological and pathophysiological processes are mediated by five molecularly distinct CHRMs labeled 1-5 [47-51]. One of the structural properties of all CHRM subtypes is a long third intracellular loop (i3) composed of about 160-240 amino acid residues. Prior studies have attempted to express human CHRM2 with a deletion in the central part of the third intracellular loop from Ser234 to Arg381 using various expression systems, including *E. coli *[17,18], *P. pastoris *[29], and insect cells [52].

The present study focuses on the potential of *P. pastoris *to express GPCR proteins that are adequate for structural analysis. The X-ray crystal structures of several membrane proteins, including the mammalian potassium channel [53] and the molluscan acetylcholine-binding protein [54], were successfully solved using the *P. pastoris *expression system. Insect cell-based expression systems have certain disadvantages for protein expression compared to *P. pastoris*, such as more involved and costlier culture requirements, as well as the difficulty in developing large-scale fermentation systems. Furthermore, the preparation of sufficient amounts of baculovirus for large-scale expression is time consuming. *P. pastoris *expression systems, on the other hand, are easily controlled for the optimization of growth conditions for the induction of the receptor of interest, including culture scale, temperature, time and pH value. In the present study, the quality and quantity of CHRM2 protein produced from *P. pastoris *and insect cells were compared for their potential use in structural studies of GPCRs.

## Results

### Comparison between culture conditions in P. pastoris and Sf9 insect cells during receptor overexpression

**Figure 1:**
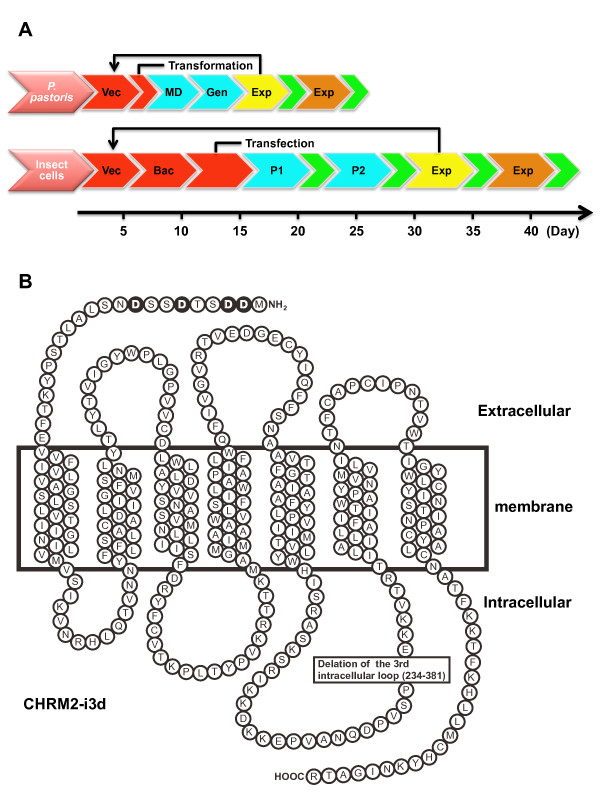
**Schema of GPCR expression in *P. pastoris *and Sf9 insect cells and human CHRM2 mutant**. The CHRM2-expressing plasmid vector was constructed (Vac.). In the *P. pastoris *expression system, the plasmid was transformed into the *P. pastoris *strain SMD1163. The transformants were inoculated onto MD plates (MD) and selected by YPD agar plates containing G418 (0.1 or 0.25 mg/mL) (Gen). In the Sf9 insect cell expression system, baculovirus was obtained by homologous recombination in *E. coli *using the Bac-to-Bac baculovirus expression system (Invitrogen) (Bac.). Baculoviruses were amplified stepwise (P1 and P2). Small- and large-scale cultures are shown in yellow and orange, respectively. Evaluation by a ligand-binding assay is represented in green (A). Four N-linked glycosylation sites in the N-terminus were eliminated by converting asparagine residues (Asn2, 3, 6 and 9) to aspartic acid (Asp). Amino acid residues 234- 381 of the third intracellular loop of the human CHRM2 were deleted (B).

In the crystal structure determination of turkey ADRB1, human ADRB2, and human ADORA2A, all three GPCRs were produced as recombinant proteins in Sf9 or High-5 insect cells. However, the yeast *P. pastoris *can grow rapidly, can reach high cell densities, and it contains the necessary elements to perform posttranslational modifications that might be essential for protein function. The use of a tightly regulated strong promoter, alcohol oxidase 1 (AOX1), which is inducible by methanol as the sole carbon source, enables the production of a large amount of recombinant GPCRs in *P. pastoris*. Figure 1A shows the temporal differences between GPCR expression using *P. pastoris *or Sf9 insect cells from expression vector construction to large-scale expression. The total time to large-scale expression was about 24-28 days in *P. pastoris *compared to about 40-45 days in Sf9 insect cells, with a temporal difference of 17-21 days between the systems. Thus, the *P. pastoris *expression system required less time to generate suitable clones for various GPCRs for crystal structural study. Figure 1B shows the construction of recombinant CHRM2 as a model protein for structural analysis using 2 expression systems.

**Figure 2:**
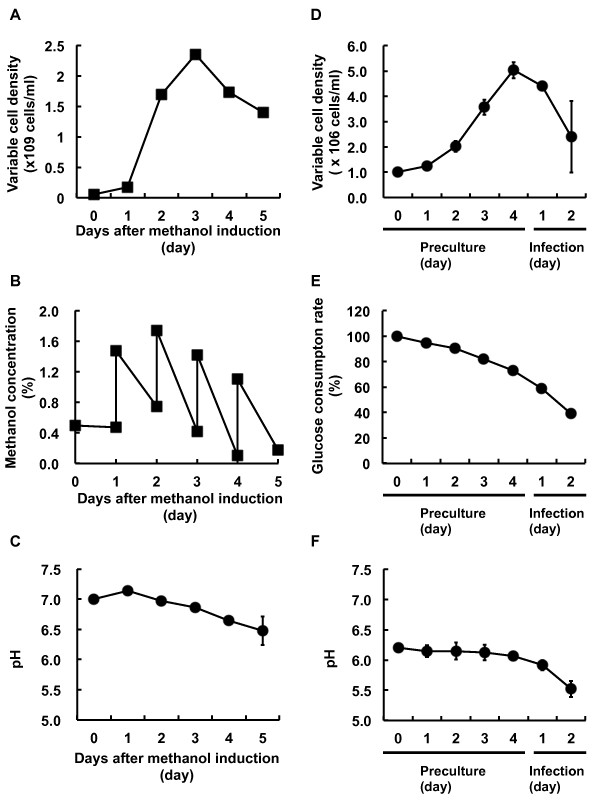
**Comparison of culture conditions in *P. pastoris *and Sf9 insect cells**. In *P. pastoris*, cell density (A), methanol concentration (B), and pH (C) were measured until 5 days after methanol induction. Cell density was estimated from OD_600_. Methanol concentration was monitored before and after methanol addition using a methanol sensor system (RAVEN Biotech Inc.). In Sf9 insect cells, cell density (D), glucose concentration (E), and pH (F) were measured during the period from preculture (4 days) to baculovirus infection (2 days). Cells were stained with 0.25% trypan blue in PBS (-) and living cells were counted with a hemocytometer.

Culture conditions such as cell density, nutrient metabolism and pH, which contribute to optimal recombinant protein production, were compared between *P. pastoris *and insect cells. *P. pastoris *transformants were inoculated and grown in BMGY medium, in which glycerol was the sole carbon source, overnight or until an OD_600 _of 2-6 was reached. The cells were then harvested by centrifugation. To induce expression of the protein of interest by methanol, the cell pellet was resuspended to an OD_600 _of 1.0, corresponding to 5 × 10^7 ^cells/ml in BMMY medium (0.5% methanol). The cell density increased up to 2.4 ± 0.3 × 10^9 ^cells/ml in the baffled shake flask until 3 days after methanol induction of the recombinant protein (Figure 2A). Methanol was added to a final concentration of 1% every 24 hr during the induction phase, maintaining the total concentration between 0.5% and 1.5% to minimize toxicity (Figure 2B). The pH in the induction medium gradually decreased, requiring 5 days for a reduction from 7.0 to 6.5 ± 0.2 (Figure 2C).

For the generation of baculovirus-infected Sf9 insect cells, baculovirus was added at a multiplicity of infections (MOI) of 2 when insect cells reached 5.0 ± 0.3 × 10^6 ^cells/ml, showing a viability greater than 95% in the cellbag using the Wave Bioreactor system. A period of 4 days was necessary to achieve the desired cell density from the seeding at 1.0 × 10^6 ^cells/ml. In contrast to the viability of *P. pastoris *during the production of recombinant CHRM2, Sf9 cell density decreased progressively after the baculovirus infection (Figure 2D). Glucose content decreased in a time-dependent manner (Figure 2E) and the pH in the culture medium remained constant at approximately 6.4 during the 4 preculture days, but rapidly decreased from 6.1 to 5.5 ± 0.1 in the 2 days after baculovirus infection (Figure 2F). The diameter of the Sf9 insect cells, which can be used as an indicator of viral infection, increased by >1 μm after 2 days of viral infection compared with uninfected Sf9 insect cells (see Fig. S1 in additional file 1).

### Optimization of CHRM2 expression parameters in P. pastoris

**Figure 3:**
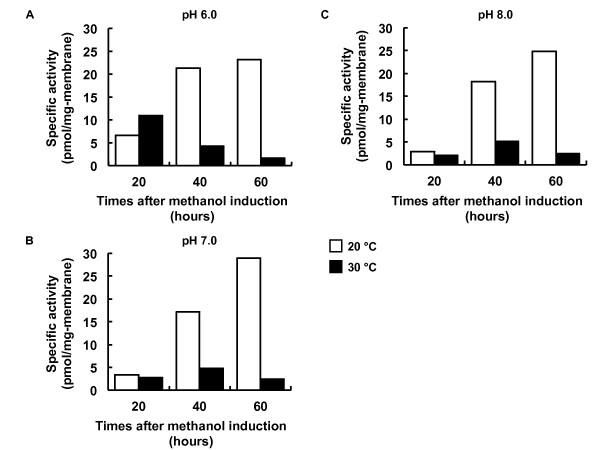
**Optimization of *P. pastoris *culture conditions**. *P. pastoris *was cultured in various conditions and the expression levels of CHRM2 were estimated by specific ligand binding assay. To find the optimal growth conditions, the pH of the medium at 6.0 (A), 7.0 (B) and 8.0 (C), the induction temperature (20 or 30°C), and expression times (20, 40 and 60 hr) were evaluated in all possible combinations.

The optimization of expression conditions, including induction time, temperature and pH, can be controlled in small-scale *P. pastoris *cultures during methanol induction for the production of the highest levels of the protein of interest. Whereas the scaling-up of the culture system in Sf9 insect cells decreased the production of CHRM2 (data not shown), the expression levels of CHRM2 in *P. pastoris *increased in correlation with the scale-up of the cell culture [29]. Specific binding of [^3^H]QNB continued to increase up to 60 hr at 20°C in pH 6.0, 7.0 and 8.0 (Figure 3A, B and 3C). The expression levels of CHRM2 at 30°C were lower than those at 20°C under most conditions (Figure 3A, B and 3C). The pH of the culture medium did not affect the expression levels. The highest specific activity (28.9 pmol/mg of membrane protein) was obtained under optimized conditions (60 hr, pH 7.0, 20°C).

### Quantity and quality of CHRM2 expressed in P. Pastoris and Sf9 insect cells

**Figure 4:**
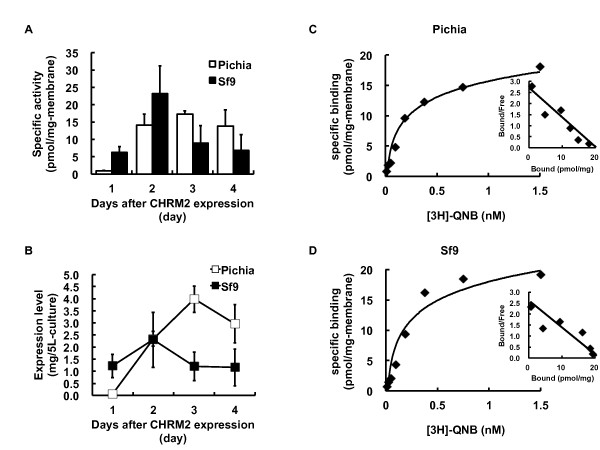
**Functional comparison of *P. pastoris *and Sf9 insect cell expression systems**. The specific activities of CHRM2 from *P. pastoris *and Sf9 insect cells are shown (A). CHRM2 expression was induced by the addition of methanol in *P. pastoris *and by the infection of baculovirus in Sf9 insect cells. The total CHRM2 protein (mg) expressed from 5 L-cultures of *P. pastoris *and Sf9 insect cells was assessed by a specific ligand binding assay (B). To compare the quality of CHRM2 from *P. pastoris *(C) and Sf9 insect cells (D), biochemical properties were evaluated by Kd and Bmax values from saturation curves and scattered plots.

The preparation of large quantities of CHRM2 possessing the appropriate ligand binding activity or affinity is necessary for structural studies. A comparison of the quality and quantity of CHRM2 expressed through large scale cultures of *P. pastoris *and Sf9 insect cells was performed by competition binding assays with [^3^H]QNB (Figure 4). A maximum specific activity was reached at 17.2 ± 1.0 pmol/mg of membrane in *P. pastoris *on day 3 after methanol induction and the high expression level was maintained until day 4 (Figure 4A). In contrast, the maximum specific activity of CHRM2 in Sf9 insect cells was obtained at 23.2 ± 8.0 pmol/mg of membrane on day 2 after baculovirus infection, followed by a progressive decrease (Figure 4A). The amount of CHRM2 expressed in *P. pastoris *gradually increased up to day 3 (Figure 4B). A total of 4 mg of CHRM2 was expressed from 5 L-culture medium on day 3. In Sf9 insect cells, 2.3 ± 1.1 mg of CHRM2 was the maximum amount expressed from 5 L-culture medium on day 2, which then decreased to approximately 1 mg/5 L-culture. Based on their maximum expression levels, CHRM2 was expressed at an almost 2-fold higher level in *P. pastoris *than in Sf9 insect cells per 5 L-culture medium (Figure 4B). High cell density in *P. pastoris *enabled the production of higher quantities of CHRM2 compared to the amounts produced in Sf9 insect cells.

To compare the quality of the CHRM2 expressed in *P. pastoris *to that produced in Sf9 insect cells, the Kd values for the binding of [^3^H]QNB to CHRM2 in both expression systems were calculated from Scatchard plots of the saturation binding experiments (Figure 4C and 4D). A representative figure of the saturation binding assay and the Scatchard plot from *P. pastoris *are shown in Figure 4C. The Bmax value was 33.5 ± 7.08 pmol/mg of membrane protein and the Kd value was 101.14 ± 15.07 pM. A representative figure of the saturation binding assay and the Scatchard plot from Sf9 insect cells are shown in Figure 4D. The Bmax value was 27.17 ± 5.57 pmol/mg-membrane and the Kd value was 86.23 ± 8.57 pM. These results indicated that the CHRM2 obtained from both expression systems had a nearly identical quality.

### Solubilization and purification of CHRM2 expressed in P. Pastoris

**Figure 5:**
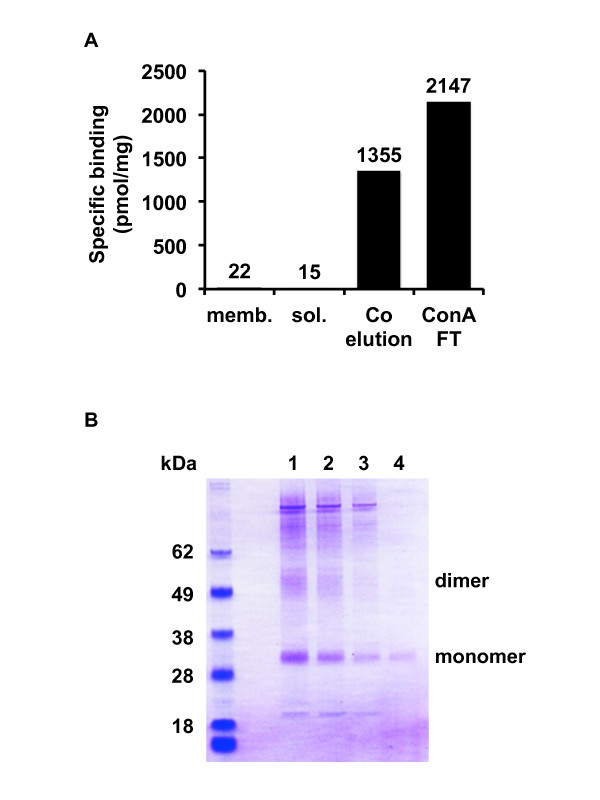
**Solubilization and purification of CHRM2 from *P. pastoris***. In each step of the purification process, CHRM2 was subjected to a specific binding assay (A) and CBB staining after SDS-PAGE (B). The specific binding assay was performed using the membrane fraction (memb.), solubilized fraction (sol.), TALON elution fraction (Co elution), and ConA flow-through fraction (ConA FT). Lanes 1-3 indicate fraction numbers 1-3 of the TALON elution. Lane 4 indicates the ConA flow-through fraction.

In a GPCR structural study, it is important to determine whether the GPCRs are able to be solubilized and purified. Therefore, we attempted the solubilization and purification of CHRM2 from *P. pastoris *(Figure 5). The purity of CHRM2 from *P. pastoris *progressively increased with each purification step. The specific activity of CHRM2 after ConA flow-through reached >2000 pmol/mg of protein, and was approximately 100 times higher than that of CHRM2 in the membrane (Figure 5A). CHRM2 purified by TALON metal affinity resin appeared as dimerized CHRM2, and impurities were identified in the SDS-PAGE and Coomassie Brilliant Blue (CBB) stain (Figure 5B). However, after ConA agarose purification, CHRM2 was well purified and observed as a single monomer band. These results showed that CHRM2 from *P. pastoris *could be solubilized and purified to the same extent as protein from Sf9 insect cells [52]. Impurities were removed at every purification step; however, purification with ConA agarose particularly removed a large amount of impurities (Figure 5B).

### Comparison of other GPCRs expressed in P. Pastoris and Sf9 insect cells

To show that many GPCRs can be isolated successfully from *P. pastoris *for crystal structural analysis, we investigated the specific binding activities of 5 mammalian GPCRs expressed in *P. pastoris *and Sf9 insect cells (Table 1). Human ADORA2A, tachykinin receptor 2 (TACR2), dopamine receptor D2 (DRD2), angiotensin receptor type 2 (AGTR2), and rat TACR2 were expressed in *P. pastoris *and Sf9 insect cells similar to CHRM2. Each GPCR was subjected to a binding assay using radioisotope-labeled specific ligands.

**Table 1:** Comparison of the specific binding activity in other GPCRs expressed from *P. pastoris *and Sf9 insect cells system

GPCR	*P. pastoris*	Sf9	Ligand
hADORA2A	220.81 ± 17.05	239.23 ± 47.12	ZM241385^a)^
hTACR2	61.96 ± 9.83	9.41 ± 2.46	SR48968^a)^
hDRD2	7.75 ± 1.54	5.98 ± 1.17	Spiperone^a)^
hAGTR2	3.69 ± 0.42	1.66 ± 0.08	Angiotenin^b)^
rTACR2	36.60 ± 12.99	14.2 ± 5.41	SR48968^a)^

All GPCRs were expressed under small-scale culture conditions. Each membrane was prepared from cells, and the specific binding assay was determined using radioactive-labeled specific ligand respectively. hADORA2A, human adenosine A2a receptor; hTACR2 and rTACR2, human and rat tachykinin receptor 2, respectively; hDRD2, human dopamine receptor D2; hAGTR2, human angiotensin receptor type 2. a, antagonist; b, agonist. The data are presented as pmol/mg-membrane and mean ± S.D. (n = 3 each).

For ADORA2A, the specific activity was extremely high (>200 pmol/mg of membrane) compared with the other GPCRs. However, there was no difference in activity between ADORA2A expressed in *P. pastoris *or Sf9 insect cells. For human and rat TACR2, the specific activities for GPCRs expressed in *P. pastoris *were 2.5-6.5 times higher than those expressed in Sf9 insect cells. The specific activities of human DRD2 and AGTR2 from *P. pastoris *were 1.3-2.2 times higher than those expressed in Sf9 insect cells. These results suggest that *P. pastoris *is a suitable host for GPCR structural studies comparable to Sf9 insect cells.

## Discussion

Insect cells such as Sf9 and High5 are the preferred expression hosts for the determination of the crystal structure of GPCRs [40-42,55]. However, the use of *P. pastoris *as a host system for the expression of GPCRs offers several advantages. *P. pastoris *is capable of expressing heterologous genes at high levels under the control of the strong and tightly regulated AOX1 promoter, and has already been used as an effective host for the X-ray crystallographic studies of the Kv channel [53,56,57]. This methylotrophic yeast combines several advantages of both prokaryotic and animal cell expression systems. It is readily amenable to genetic manipulation, it can be easily grown to high cell densities using minimal media, and it possesses the necessary elements to introduce eukaryotic posttranslational modifications. The present study compared Sf9 insect cells and *P. pastoris *as expression systems for the production of GPCRs for crystallization and structural analysis. As shown in Figure 2, culture conditions including cell density, nutrient metabolism and pH were monitored during the production of CHRM2 as a model protein in Sf9 insect cells and *P. pastoris*.

The cell density of *P. pastoris *increased up to day 3 during methanol induction, and then began to decrease as the culture was starved of dissolved oxygen (dO_2_) and nutrients (Figure 2A). As a result, the concentration of *P. pastoris*-expressed CHRM2 was highest on day 3 after methanol induction (Figure 4B). On the other hand, the highest expression level in Sf9 insect cells, which was obtained on day 2 (2.0 mg/5L-culture), amounted to one half of the maximum expression in *P. pastoris *(4.0 mg/5L-culture) (Figure 4B). The viability of insect cells decreased by 50% in two days after baculovirus infection (Figure 2D), whereas the cell density in *P. pastoris *increased until the maximum expression level was reached on day 3 (Figure 2A). Western blotting to detect CHRM2 revealed significant proteolytic degradation of the expressed protein in Sf9 insect cells [58]. Proteolytic degradation in virus-infected insect cells can be attributed to the action of carboxyl and cysteine proteases [59]. Addition of aprotinin and pepstatin A, which are inhibitors of carboxyl and cysteine proteases, respectively, to a virus-infected insect cell culture increased the yield of the infectious bursal disease virus structural protein nearly 2-fold [60]. The viability in a virus-infected cell culture might be associated with the yield of functional CHRM2 obtained in Sf9 insect cells. An expression level greater than 1 mg/L-culture is the preferred level for purification and crystallization experiments. Warne et al. expressed more than 1 mg of unpurified mutant turkey ADRB1 per liter of culture using the baculovirus system [13] and improved the expression level (numbers of functional receptors/mg of membrane) to 360 pmol/mg solubilized membrane protein, in addition to enhancing the total expression level. These results suggest that not only substantial quantities but also expression levels are critical factors for structural analysis.

To determine whether the recombinant CHRM2 expressed in *P. pastoris *and Sf9 insect cells was different in quality, a Scatchard analysis was performed from a saturation [^3^H]QNB binding experiment to determine a dissociation constant (Kd). The Kd values measured in the present study from *P. pastoris *(101.14 ± 15.07 pM) and Sf9 insect cells (86.23 ± 8.57 pM) were comparable to those reported by previous studies. A Kd value of 25.7 pM and a Bmax value of 5.55 pmol/mg protein were reported for Sf9 insect cells [17]. A Kd value of 113.7 pM and a Bmax value of 51.2 pmol/mg were reported for *P. pastoris *[29], suggesting the absence of significant differences in ligand binding activity between the two systems. The expression of cell surface molecules was monitored by flow cytometry to assess the production of properly folded proteins in the baculovirus expression system [61]. Cell surface expression of CHRM2 may result in differences between receptors produced in *P. pastoris *and Sf9 insect cells. However, surface expression, G-protein coupling and second messenger generation other than saturation ligand binding should be investigated to evaluate the sample grade for structural analysis.

As shown in Figure 3, the conditions used for the expression of CHRM2 in *P. pastoris *using a flask method were optimized based on a previously published method [29]. Flask cultures enable the application of several expression conditions in parallel and require limited equipment compared to bioreactor-based systems [27]. The addition of methanol to the *P. pastoris *culture every 24 hr allows the control of the amount of nutrients added to the culture (Figure 2B). In the insect culture system, 35% of the glucose added as a nutrient to the cell culture medium had already been consumed at the time of baculovirus infection (Figure 2E). However, methanol concentration must be kept within a narrow range because excess formaldehyde produced by the oxidation of methanol by AOX1 can accumulate to cytotoxic levels [62]. The optimum pH range for stabilization and activity differs for each GPCR and should therefore be adjusted for each receptor during induction. The *P. pastoris *expression system enables the adjustment of pH, as shown in Figure 3, which shows that there were no variations in pH values during the first three days after induction (Figure 2C).

It is important that GPCRs expressed from *P. pastoris *are evaluated for their solubilization and purification efficacy compared to proteins expressed from insect cells. The specific binding activities of CHRM2 and 5 other GPCRs expressed from *P. pastoris *and Sf9 insect cells were investigated (Figure 4 and Table 1). The specific activities of these GPCRs from *P. pastoris *were higher than those from Sf9 insect cells. Functional CHRM2 expressed from *P. pastoris *was solubilized and purified similar to CHRM2 expressed from Sf9 insect cells [52] (Figure 5).

Although previous studies have used *P. pastoris *as a GPCR expression host [24-29], to our knowledge this study is the first report to compare GPCRs expressed from *P. pastoris *directly with those expressed from Sf9 insect cells. The results of this study strongly suggest that many GPCRs, including CHRM2, can be expressed from *P. pastoris *with quality and quantity equal to those expressed from Sf9 insect cells. We think that GPCRs expressed from *P. pastoris *will be usable for crystal structural analysis.

## Conclusion

The present study identified several advantages of *P. pastoris *over insect cells as an expression system for the production of GPCRs for structural studies. Compared to insect cells, the yield of CHRM2 expressed in *P. pastoris *was higher and the receptors produced by *P. pastoris *were also functional. The optimization of culture conditions, including pH, induction time and temperature, was essential to obtain an adequate GPCR expression level in *P. pastoris*. Flask culture systems allow the optimization of several conditions in parallel, and the optimized conditions can be applied to bioreactor cultures, which yield higher cell densities than flask cultures [27]. The *P. pastoris *expression system is not only useful for functional and structural studies but also for the screening of more stable mutants with high expression levels, such as N-terminal, C-terminal, and/or third intracellular loop truncation, replacement of the third intracellular loop by T4 lysozyme, N-linked glycosylation deletion and other mutations.

## Methods

### Materials

The pPIC9K vector and *P. pastoris *strain SMD1163 were purchased from Invitrogen. Sf9 insect cells and recombinant CHRM2 baculovirus were prepared as described previously [52]. IPL-41 insect medium was purchased from AppliChem. ESF-921 insect medium was purchased from Expression Systems. SF900II insect medium, tryptose phosphate, TC extract, Pluronic F-68 and fetal bovine serum were purchased from Invitrogen. Penicillin-streptomycin mixture was purchased from Wako. [^3^H]QNB (quinuclindinyl benzilate, 1.74 TBq/mmol) were obtained from GE Healthcare. Atropine was purchased from Sigma. GF/F glass fiber filters were purchased from Whatman and protease inhibitor cocktail tablets (Complete) were purchased from Roche Diagnostics.

### Construction of CHRM2 encoding vector for P. pastoris

CHRM2 cDNA was subcloned into the pPIC9K expression vector and linearized using the restriction enzyme PmeI. The linearized vector was transformed into the *P. pastoris *strain SMD1163 by electroporation (1500 V, 25 μF, and 600 Ω) using a Gene Pulser I (Bio Rad). Clone selection was performed as previously described [63,64]. In brief, recombinant His^+ ^clones were selected on MD agar plates (1.34% (w/v) yeast nitrogen base without amino acids, 2% (w/v) dextrose, 0.00004% (w/v) biotin, and 1.5% (w/v) agar). To select for multicopy transformants, His^+ ^clones were grown on G418-YPD agar plates (1% (w/v) yeast extract, 2% (w/v) peptone, 2% (w/v) dextrose, 2% (w/v) agar, and 0.1 or 0.25 mg/ml G418). Representative clones exhibiting resistance to G418 were tested for recombinant protein production by specific ligand binding assays using [^3^H]QNB. The selected transformants were stored as glycerol stocks at -80°C.

### Small scale culture for P. pastoris

Glycerol stocks of the transformants were inoculated onto YPD agar plates containing 0.1 mg/ml G418. For receptor production, cells were precultured in 5 ml BMGY medium (1% (w/v) yeast extract, 2% (w/v) peptone, 1.34% (w/v) yeast nitrogen base without amino acids, 0.00004% (w/v) biotin, 1% (w/v) glycerol, and 0.1 M phosphate buffer at pH 6.0) at 30°C with shaking at 250 rpm until an OD_600 _of 2-6 was reached. Induction of CHRM2 expression was conducted in 5 ml BMMY medium (1% (w/v) yeast extract, 2% (w/v) peptone, 1.34% (w/v) yeast nitrogen base without amino acids, 0.00004% (w/v) biotin, 1% (v/v) methanol, and 0.1 M phosphate buffer at pH 6.0, 7.0, or 8.0) containing 0.04% (w/v) histidine and 3% (v/v) DMSO at 20 or 30°C from an initial OD_600 _of 1. The procedure used for *P. pastoris *culture was described previously [29]. After a 20, 40, or 60 hour induction, cells were harvested at 4,000 g for 15 min, washed once with ice cold water, and frozen or immediately used for membrane preparation.

### Large scale culture for P. pastoris

Single *P. pastoris *colonies from high expressing clones were selected on YPD plates containing 0.1 mg/ml G418. Cells from a single colony were used to inoculate 200 ml of BMGY medium. The culture was grown overnight at 30°C to an OD_600 _of 2-6. A total of 500 ml of BMGY was inoculated with 100 ml of the starter culture and grown for 4 hr to an OD_600 _of 5-10. The cells were spun down at 4,000 g for 15 min, the cell pellet was washed with double distilled water, and then the cells were spun down. The cell pellet was resuspended in 500 ml BMMY to an OD_600 _of 1. The culture was incubated for 1-4 days at 20°C with shaking at 250 rpm, and then 25 ml of 20% methanol was added to the cell culture to maintain a final methanol concentration of 1% every day until the end of the culture period.

### Small scale culture for Sf9 insect cells

Sf9 insect cells were maintained in IP-41-based medium or ESF-921 medium each containing 3.5, 5 or 7% heat-inactivated fetal bovine serum. Preparation of culture medium was described previously [65]. Briefly, complete culture media were mixed at a ratio of 70% IPL-41 medium to 30% SF900II serum free medium and supplemented with 0.1% pluronic F-68 and 3.5 or 7% FBS. The small scale culture was carried out to transfer to large scale culture using a wave bioreactor (GE Healthcare). Sf9 insect cells were cultured in Erlenmeyer flasks with the 125 rpm at 27°C culture conditions and passaged every 3 or 4 days.

### Large scale culture for Sf9 insect cells

Sf9 insect cells were prepared at a density of 1.0 × 10^6 ^cells/ml and suspended in 5 L of the IPL-41/SF900 II complex media or ESF921 insect media. Media containing Sf9 insect cells were transferred into the CELLBAG 22 L/O (GE Healthcare) [66,67] and cultured for 4 days with the following culture conditions: 20 rpm, 8.5° of rocking angle, 30% O_2_, 0.25 L/min of air flow rate, and 27°C. After 4 days, 200~300 ml of baculovirus stock (approximate multiplicity of infection (M.O.I) = 2) and 700~800 ml of IPL-41/SF900 II complex media were transferred into the CELLBAG (final culture volume = 6 L) and infected for 2 days under the following infection conditions: 22 rpm, 8.5° of rocking angle, 50% O_2_, air flow rate, 0.25 L/min, and 27°C. Two days later, a fraction of the cells was harvested for the binding assay and the remaining cells were centrifuged at 6,000 × g for 10 min and harvested. The cell pellet was washed with 250 ml of Phosphate Buffered Saline without calcium chloride and magnesium chloride (PBS(-)) and resuspended with 100 ml of PBS(-) containing a protease inhibitor cocktail tablet (Roche). Cells were quick frozen in liquid nitrogen and stored at -80°C.

### Preparation of the cell membrane

The preparation of cell membranes from *P. pastoris *was performed at 4°C or on ice. Harvested cells (1 g of wet weight) were suspended in 4 ml of lysis buffer (50 mM sodium phosphate buffer, pH 7.4, 100 mM NaCl, 5% (v/v) glycerol, and 2 mM EDTA) containing a cocktail of protease inhibitors (1 tablet/100 ml lysis buffer). After the addition of 0.5 mm glass beads to the cell suspension, yeast cells were disrupted by vortexing at 4°C for 2 hr. Lysis efficiency was evaluated using a light microscope and was usually more than 80%. Intact cells and cell debris were separated from the membrane suspension by low speed centrifugation (3,000 g, 5 min and 4°C). Membranes were snap-frozen in liquid nitrogen and stored at -80°C.

For the preparation of membranes from insect cells, Sf9 insect cells were centrifuged at 1,500 g for 10 min at 4°C. The pellet was washed with PBS(-), then resuspended in 100 ml of hypotonic buffer containing 10 mM HEPES at pH 7.5, 20 mM potassium chloride, 10 mM MgCl_2_, and protease inhibitor cocktail, followed by Dounce homogenization to resuspend the membranes. Insect cell membranes were centrifuged at 100,000 g for 30 min and the pellets were resuspended in 10 mM HEPES at pH 7.5, 10 mM magnesium chloride, 20 mM potassium chloride, and 40% glycerol, and then flash-frozen in liquid nitrogen and stored at -80°C until further use.

### Solubilization and purification of CHRM2

Membrane suspensions of the CHRM2 were solubilized in solubilization buffer containing 20 mM HEPES, pH 7.0, 500 mM NaCl, 20% glycerol, 1% n-dodecyl-β-D-maltopyranoside (DDΜ) (Affymetrix), 0.2% cholesterol hemisuccinate (CHS) (Sigma), and protease inhibitor cocktail at 4°C for 2 hr. The insoluble materials were pelleted by ultracentrifugation at 200,000 g for 1 hr at 4°C. TALON metal affinity resin (Clontech) equilibrated in purification buffer (20 mM HEPES, pH 7.0, 800 mM NaCl, 10% glycerol, 0.05% DDM, 0.01% CHS and protease inhibitor cocktail) was added to the solubilized membranes and incubated for 2 hr at 4°C. CHRM2 protein was eluted with purification buffer containing 250 mM imidazole. ConA agarose (Seikagaku Corporation) equilibrated in purification buffer was added to the TALON-purified CHRM2 and incubated overnight at 4°C. The ConA agarose flow-through fraction was harvested. The purified CHRM2 protein was subjected to SDS-PAGE, CBB staining, and a specific binding assay.

### Radioligand binding assay

Binding assays were conducted in polystyrol tubes using assay buffer (20 mM potassium phosphate buffer). GF/F glass filters were presoaked in 200 ml of 0.3% (v/v) polyethyleneimine. All experiments were performed in duplicate in a total volume of 200 μl. Membrane proteins were quantified using the bicinchoninic acid (BCA) method (Thermo Fisher Scientific) with bovine serum albumin as a standard. Membrane proteins (2.5 μg) were incubated for 30 min at 25°C in 200 μl of assay buffer containing 1.5 nM [^3^H]QNB for single point binding assays at saturating radioligand concentrations. Non-specific binding was obtained by incubation in the presence of an excess of the non-radioactive ligand, namely in 100 μM atropine. Bound and free ligands were separated by rapid vacuum filtration over GF/F filters. Filtration was carried out with a Brandel cell harvester at room temperature. Filters were washed three times with ice cold water. The retained radioactivity was measured on an LCS-5100 liquid scintillation counter (ALOKA).

### Experimental design and Statistical evaluation

Experiments in each group were repeated independently at least 3 times. The Kd and Bmax values were estimated using the saturation binding assay and Scatchard plots. Data are presented as the means ± standard deviation (S.D.).

## Competing interests

The authors declare that they have no competing interests.

## Authors' contributions

HA, TU and TS carried out the optimization of Sf9 expression conditions. TY-K, KI, TS and TN carried out the optimization of *P. pastoris *expression conditions. MS and HT performed the ligand binding assays. NN and TM contributed to the data analysis and interpretation. TH constructed the baculovirus vector carrying the CHRM2 gene. SI and TK designed the study and reviewed the final manuscript. All authors read and approved the final manuscript.

## Supplementary Material

Additional File 1 **Fig. S1. Morphological change of Sf9 insect cell by baculovirus infection**. Healthy, uninfected Sf9 insect cells (A) and cells 2 days after baculovirus infection (B) were mounted on slide glass and observed under bright-field microscopy. (×40) (Scale bar, 10 μm. Images were independently observed 3 times and representative images are shown. Click here for file
