# Supplementary figures and images for: Evaluation of the Pichia pastoris expression system for the production of GPCRs for structural analysis
Source: Microb Cell Fact. 2011 Apr 22;10:24. doi: 10.1186/1475-2859-10-24 (PMC3094209; doi:10.1186/1475-2859-10-24)

**A**

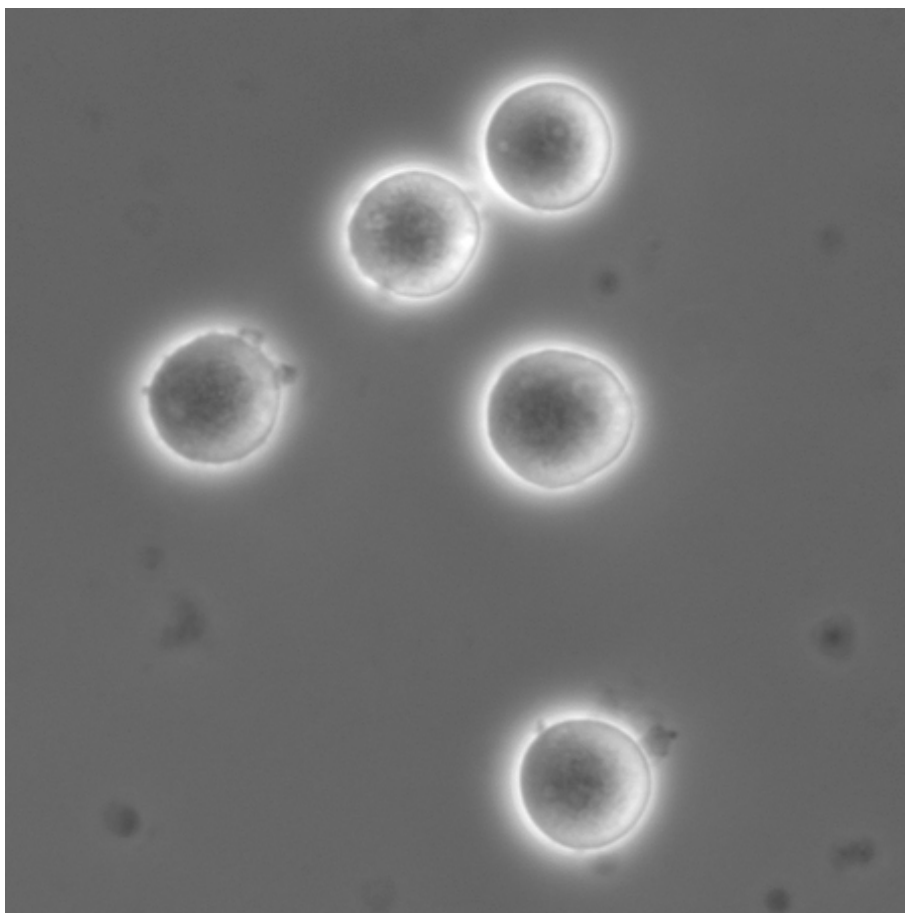

**B**

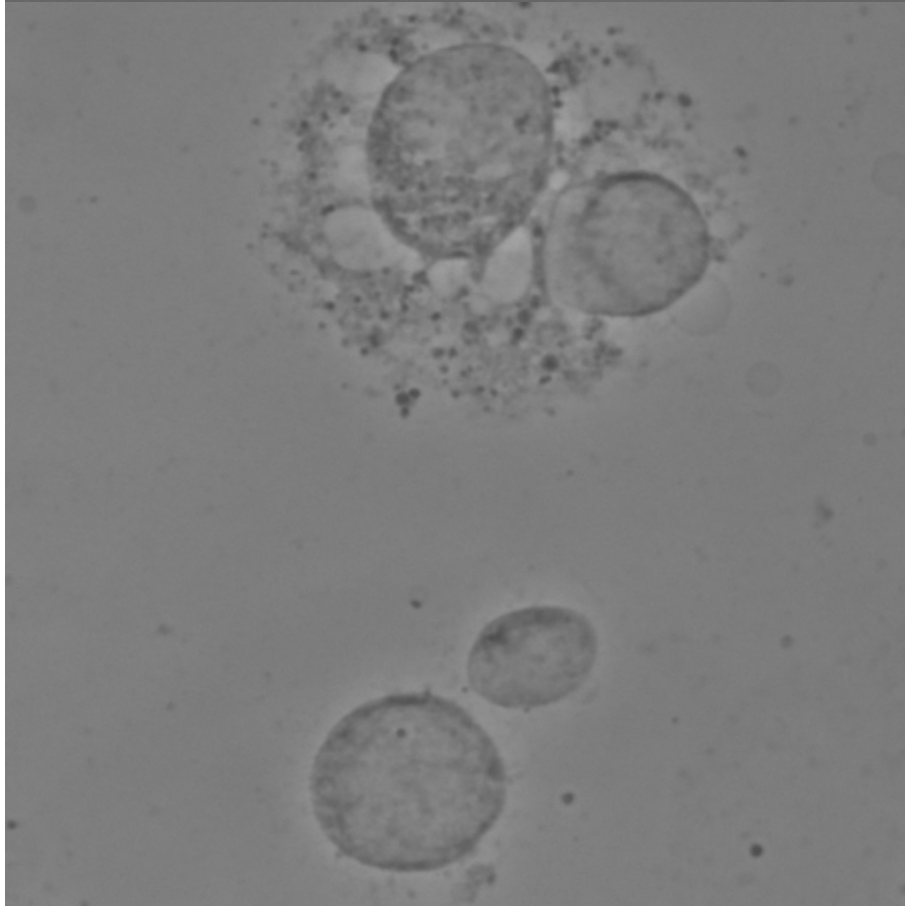

— 10  $\mu\text{m}$

Supplement: Additional File 1 — Fig. S1. Morphological change of Sf9 insect cell by baculovirus infection. Healthy, uninfected Sf9 insect cells (A) and cells 2 days after baculovirus infection (B) were mounted on slide glass and observed under bright-field microscopy. (×40) (Scale bar, 10 μm. Images were independently observed 3 times and representative images are shown. [file 1475-2859-10-24-S1.PDF]
